# Tensions in patient involvement and engagement in health research and development: a qualitative interview study with key actors

**DOI:** 10.1186/s40900-026-00915-3

**Published:** 2026-06-11

**Authors:** Kiana Kiani, Vinh Phillips Ingvason Nguyễn, Elin Siira, Catarina Wallengren, Axel Wolf, Ida Björkman

**Affiliations:** 1 Institute of Health and Care Sciences, Sahlgrenska Academy, Gothenburg University, Gothenburg, Sweden; 2 Centre for Person-Centred Care (GPCC) Sahlgrenska Academy, Gothenburg University, Gothenburg, Sweden; 3 School of Health and Welfare, Halmstad University, Halmstad, Sweden

**Keywords:** Patient involvement, Patient engagement, Key actors, Facilitators, Barriers, Health research, Health development, Person-centred healthcare

## Abstract

Patient involvement and engagement (PIE) is increasingly recognised as essential for improving health research and service design and for supporting the transition toward person-centred healthcare systems. However, the implementation of PIE remains inconsistent across health research and development globally. The aim of this study was to examine how barriers and facilitators to PIE in health research and development are understood and negotiated by different key actors. This study adopted a qualitative design. Semi-structured interviews were conducted with a diverse group of key actors, including patient representatives, healthcare professionals, health researchers, representatives from pharmaceutical and medical industry, and public authorities (*n* = 29). Participants were recruited through the European Patients’ Academy on Therapeutic Innovation (EUPATI) Sweden and had prior experience of PIE in their respective roles. The data were analysed using qualitative content analysis, as described by Graneheim and Lundman. The findings revealed barriers and facilitators related to the following themes: *Shifting culture*,* power and responsibility*; *Guidance through clear structures*; *Expectations of the patient representative role*; *Trust and communication*; and *Towards shared understanding*. The different actors’ understandings of these barriers and facilitators create tensions that need to be acknowledged and negotiated in order to drive PIE forward. We suggest that incorporating the ethical foundation for person-centred approach with narrative, partnership and shared responsibility trough documentation in PIE activities would offer a way forward, as many of the barriers and facilitators identified were related to how PIE was carried out in practice. Further studies are needed to examine how incorporation of Person-centred approach in PIE would be implemented and evaluated with the aim of strengthening collaboration and enabling more meaningful involvement.

**Supplementary Information:**

The online version contains supplementary material available at 10.1186/s40900-026-00915-3.

## Abstract (plain language summary)

Involving patients in healthcare research and development is becoming more important for creating services that better meet people’s needs. This study explored how different actors involved in health research and development understand and handle the challenges and opportunities of patient involvement. We interviewed 29 people, including patient representatives, healthcare professionals, health researchers, representatives from pharmaceutical and medical technology industry, and government authorities. Participants were recruited through EUPATI Sweden and had previous experience of patient involvement. The findings show that involvement is shaped by several challenges and opportunities. These relate to issues such as organisational structures, expectations of the patient representative role, power and responsibility between actors, trust and communication, and differences in how involvement is understood. The different actors involved understand these barriers and helpful factors in different ways, which can create tensions and misunderstandings. Such differences need to be openly discussed and worked through together in order to move PIE forward. We suggest that bringing the core ideas of Person-Centred approach into PIE activities, such as narrative, partnership and shared responsibility trough documentation, could help address many of the challenges identified, since many of them were connected to relationships and to how PIE was carried out in practice. More research is needed to explore how a Person-Centred approach can be integrated into PIE in practice and how this could strengthen collaboration and support more meaningful involvement.

**Supplementary Information:**

The online version contains supplementary material available at 10.1186/s40900-026-00915-3.

## Introduction

Patient involvement and engagement (PIE) and its implementation is increasingly recognised as essential to improving health research and development [1–3]. It forms part of a broader shift towards more democratic and person-centred health systems and is supported by policy and legislative frameworks promoting patient involvement although a clearer theoretical foundation and a more deliberate practice are still needed [4, 5]. Spanning a continuum from minimal interaction to substantive collaboration, PIE may occur across different stages of the health research process. However, its implementation remains inconsistent and characterised by lack of conceptual clarity, among other factors [1, 6–8].

Several terms are used to describe the role of patients and the public in health research and development, including involvement, engagement, and participation, and these are often used interchangeably. While the terminology varies across policy documents, guidelines, and research contexts, the concepts generally refer to different degrees of participation in healthcare decision-making and research processes [9, 10]. The different terms can be understood as representing different positions along a continuum, where involvement may reflect a minimum level of interaction, while engagement refers to more interactive and collaborative forms of participation in health research and development [1, 6, 11, 12]. This study deliberately combined the terms *patient involvement* and *engagement* (PIE) to capture experiences across the continuum of participation described in the literature.

Despite its growing prominence, PIE still faces a gap between theory and practice, driven by implicit assumptions, unclear expectations, and limited evidence of impact, underscoring the need for clearer structures and effective implementation support [9, 10, 13–15]. Attention to contextual barriers and facilitators, supportive processes and experiences of all actors involved is therefore required [13–15].

Existing research has identified a range of barriers and facilitators for PIE, as well as factors required to enhance its implementation. According to Reardon et al. 2026 [16], the outcomes of integrating a new research approach such as PIE into routine organisational practice are influenced by the characteristics of the initiative itself, the internal and external context, the individuals involved, and the processes that shape its uptake. Barriers such as unclear roles, power imbalances, communication challenges and limited institutional support may result in tokenistic involvement or hinder sustained collaboration. In contrast, trustful relationships, role clarity, capacity building and supportive organisational structures can enable more meaningful and sustainable PIE. These factors may act as either barriers or facilitators depending on how they are recognised and addressed by the actors involved [16–19]. Approaches to PIE also vary across context and remain inconsistently implemented, reflecting differences in understanding, infrastructure and expectations, as well as ongoing challenges in generating evidence for participatory approaches [20–22].

Despite strong political ambitions and policy commitments, PIE is often understood and enacted differently across different contexts and by different actor groups, including patient organisations, healthcare professionals, health researchers, the pharmaceutical and medical technology industries, and public authorities. Systematic reviews show that professionals, service users and organisational leaders hold divergent expectations regarding the purpose, scope and outcomes of involvement, which may lead to tensions or gaps between policy rhetoric and everyday practice [19–21]. The complexity of PIE is further amplified by the involvement of actors operating within distinct regulatory, professional and organisational contexts, as well as differences in knowledge traditions and regulatory demands. Institutional logics and resources may shape who participates, with what influence, and how involvement is interpreted, resourced and enacted in practice [1, 2, 20–25]. This makes it challenging to define concepts precisely and to understand their distinct implications, which may in turn contribute to a limited understanding of the structural factors influencing the implementation of PIE [24–26].

In the Nordic countries, as in other countries, PIE has increasingly been integrated into health research and development, but its implementation is still evolving [22, 23]. In Sweden, patient rights have been strengthened, as exemplified by the introduction of the Patient Act (SFS 2014:821) in 2015, which reinforced patients’ rights to information, consent and participation in care decisions and formed part of an earlier policy shift that supported this development. According to the act, healthcare professionals are required to involve patients in decisions regarding treatment and care, and regional authorities have introduced initiatives such as Coordinated Individual Plans (CIP) and digital tools to support patient empowerment alongside national guidelines and follow-up systems [26]. Beyond individual care, PIE have also become increasingly prominent in health research and development. PIE has been progressively institutionalised through patient councils and the involvement of patient organisations in evaluation, quality improvement and service development. Previous research shows that such structures can enable patients to influence both service improvement and management-level decisions, and patient and interest organisations have gained a more visible role in shaping health policy and care development in Sweden [14, 27].

Despite a growing body of research on barriers and facilitators for PIE, as well as potential approaches to support its development and evaluation, the implementation and evaluation of PIE remain fragmented [1, 2, 15]. There is a need to explore whether additional factors beyond those identified in previous research can be highlighted to better support the implementation of PIE. The present study is situated in a Swedish context and examines how barriers and facilitators related to PIE are understood and negotiated among patient representatives, health researchers, public authority representatives, healthcare professionals, and representatives from the pharmaceutical and medical technology industry. These actors represent different organisational and system levels of decision-making and governance. Drawing on interviews, the study provides insight into how barriers and facilitators are understood and negotiated by the actors involved, as well as the tensions that emerge from their different understandings of these factors. These findings can inform more effective and sustainable approaches to PIE.

## Method

### Aim

The aim of this study was to examine how barriers and facilitators to PIE in health research and development are understood and negotiated by different key actors.

### Study design

This qualitative interview study is part of a PhD project developed in collaboration between the University of Gothenburg’s Centre for Person-Centred Care (GPCC) and the European.

Patients’ Academy on Therapeutic Innovation (EUPATI) Sweden [28]. To examine how barriers and facilitators to PIE were understood and negotiated by different key actors, semi-structured interviews were conducted and analysed using qualitative content analysis – an approach well suited to exploring how participants understand and make sense of complex phenomena [29–31]. The analysis followed the approach described by Graneheim and Lundman [29, 30]. To ensure comprehensive reporting, the study was reported in accordance with the Consolidated Criteria for Reporting Qualitative Research (COREQ) guidelines in Additional file 1 [32].

### Patient and public involvement

Patient and public involvement (PPI) was embedded throughout the different phases of the PhD project. One patient researcher (JB) affiliated with GPCC and a EUPATI Fellow contributed to the overall planning and development of the PhD project. The research questions for the PhD project were refined in collaboration with senior researchers at GPCC. JB also provided advisory input in developing the interview guide for this study.

In addition, a patient co-researcher (VPIN) and member of the GPCC Patient Council was involved in data analysis, interpretation, and manuscript development. VPIN actively contributed to the analysis, theme development and manuscript review to ensure relevance from a patient perspective. Drawing on lived experience, the patient co-researcher helped contextualise the findings and bridge perspectives between researchers and patients. He also contributed to the discussion and conclusions, thereby strengthening the study’s credibility and relevance. The aim of patient and public involvement (PPI) in this study was to integrate the patient perspective throughout the research process, from the formalisation of the PhD project to data analysis and the final manuscript.

Patient and public involvement in this study is reported in accordance with the Guidance for Reporting Involvement of Patients and the Public (GRIPP2) checklist [33]. A completed GRIPP2 short form is included as Additional file 2.

## Settings

This study was conducted within the context of EUPATI Sweden, an organisation that aims to promote increased patient involvement in research and in the development of new therapies, medical-technology solutions, and life-science innovation [28]. EUPATI Sweden was established in 2021 as a national project led by the Swedish Disability Rights Federation and funded by the Swedish Inheritance Fund. Representatives from 15 patient organisations, healthcare professionals, academia, health agencies, and the pharmaceutical and medical technology industry have been involved with EUPATI-Sweden in various capacities. For the purpose of increasing patient involvement in research and development, EUPATI Sweden has developed a cost-free, publicly accessible digital educational platform designed to strengthen patient and public knowledge in areas such as research, regulation, and ethics. Since 2024, EUPATI Sweden has operated as a nonprofit association representing patients, academia, industry, and the public sector, functioning as a neutral platform for education and collaboration. In 2025, the Swedish government provided additional funding to support the development of a long-term national collaboration platform. Participants in the present study were recruited within this organisational and educational setting.

## Participants

### Recruitment and sampling

Participants were recruited through EUPATI Sweden on two separate occasions to include key actors representing different roles relevant to PIE. The first recruitment occurred in connection with the launch of EUPATI Sweden’s educational platform in Sweden in the spring of 2023. Before the official launch of the platform, EUPATI Sweden engaged 30 patient representatives to test a pilot version of the platform. All participants were thereafter invited by the research team to take part in an interview following their testing of the platform. Of the 30 participants, 27 expressed interest, and 14 ultimately confirmed their participation and were interviewed.

The second recruitment was conducted in autumn 2023 through a survey distributed to all members of EUPATI Sweden (*n* = 200). The survey formed part of a separate study, and its findings are not included in this study. In the final survey item, participants were asked whether they would be interested in participating in the present interview study. Of the 200 members, 50 expressed interest in participating. To ensure diversity among key actor groups, a purposive sampling strategy was applied [49]. This resulted in a final pool of 18 individuals who met the inclusion criteria – namely, affiliation with one of the following groups: public authorities, healthcare professionals, representatives from the pharmaceutical/medical technology industry, or health researchers. These 18 individuals were invited via email to take part in individual interviews. Fifteen accepted and were subsequently interviewed.

### Participant characteristics

The study participants (*n* = 29) included patient representatives (*n* = 14), health researchers (*n* = 3), public authority representatives (*n* = 2), healthcare professionals (*n* = 4) and representatives from the pharmaceutical/medical technology industry (*n* = 6). In this study, all participants are referred to as key actors. See Table 1 for an overview of the participants’ characteristics.

**Table 1:** Overview of participants

Key actors	*n*	Men	Women	Non binary	Mean age	Age range
**First recruitment**						
Patient Representative	14	3	10	1		
**Second recruitment**						
Health researcher	3	1	2			
Public authority representative	2	2	0			
Healthcare professional	4	0	4			
Pharmaceutical/medical technology industry representative	6	2	4			
	29	8	20	1	50	32–74

## Data collection

Data were collected through semi-structured interviews following Kvale and Brinkmann [34]. The interviews were conducted remotely online via Zoom – an approach which facilitated recruitment of geographically distributed participants. A pilot interview was conducted to test the clarity and relevance of the interview guide and to refine the interview technique. Only minor adjustments in wording were made thereafter. The interview guide addressed participants’ understanding of PIE, collaboration with other actors, perceived power to influence PIE, and experienced barriers and facilitators (see Additional file 3).

A total of 29 individual interviews were conducted by EBW (*n* = 6) and KK (*n* = 23) between March 2023 and January 2024. The interviews lasted 24–56 min (mean 36 min) and were audio-recorded and transcribed verbatim. Although moderate in length, the interviews generated rich information. The participants were experienced and knowledgeable in relation to PIE, which enabled in-depth discussions. Seven interviews were transcribed manually by the research team as part of the learning process for the junior researchers (KK and EBW) and to enhance familiarity with the data. The remaining interviews were transcribed by a professional transcription service in accordance with standard university practice.

Recordings and transcripts were stored on an encrypted, password-protected computer in accordance with the University of Gothenburg’s data protection guidelines and GDPR [35]. Access was restricted to the research team, and all identifying information was anonymised during transcription.

### Data analysis

The interviews were analysed using inductive qualitative content analysis, as described by Graneheim and Lundman [29, 30]. A qualitative content analysis approach was considered appropriate as the aim of the study was to explore how different actors understand and interpret barriers and facilitators related to patient involvement and engagement. The analysis focused on latent content, enabling interpretation of underlying meanings beyond the manifest text. The research team consisted of one junior researcher (KK), four senior researchers (IB, CW, ES, AW), and one patient co-researcher (VPIN).

The analysis began with individual readings of the transcripts by KK, IB and VPIN to gain an overall understanding of how collaboration was understood and negotiated by the different actors. Meaning units (*n* = 1816) relevant to the research aim were identified by KK, IB and VPIN, and subsequently coded using Microsoft Excel as a practical tool to organise and structure the data during the analytic process. Initially, 94 codes were generated and subsequently grouped into 50 codes. Codes with shared meanings were then grouped and interpreted into broader thematic patterns, resulting in five themes. A compilation of the codes identified within each theme is presented in Additional File 4. Meaning units are segments of text, such as words, sentences or paragraphs that relate to a shared central meaning. Three interviews were initially coded jointly at different times during the analysis process to establish a shared analytic approach, followed by individual coding of the remaining transcripts. Team discussions were used to enhance reflexivity and actively explore divergent interpretations. Any meaning units that did not fit emerging codes or themes were revisited in relation to the original transcripts and the study aim. Alternative interpretations were discussed to ensure that the analysis remained grounded in the data. Guided by the aim of the study, the researchers moved iteratively between the whole and the parts of the text (de-contextualization and re-contextualization) throughout the analysis process [36]. Codes were subsequently sorted into themes through collaborative discussions involving all researchers (KK, VPIN, IB, ES, CW and AW). This process of researcher triangulation [29, 37] aimed to strengthen analytic rigour by incorporating multiple perspectives and reducing the risk of premature closure of interpretation. Table 2 illustrates the analytic process, providing an example of how a meaning unit was assigned a code and subsequently grouped into a theme.

**Table 2:** Examples of meaning units, codes and themes

Meaning unit	Code	Theme
*“The most power…If we look at it in terms of the actual healthcare meetings*,* it has to be the doctors. But actually*,* I think the nurses have the greatest opportunity to promote participation…Then the power to grant participation lies with let’s say*,* both*,* government bodies or SKR*,* for example… ” Code 2*,* Patient representative*	Ambiguity in power relations in the context of collaboration	Shifting Culture, Power and Responsibility
*“Well*,* if you don’t have it*,* and it doesn’t become part of the routine work*,* then it kind of gets hard to – if we’re supposed to reinvent the wheel each time…I think it’s an important part – to work systematically…” Code 25*,* Public authority representative*	The importance of a systematic approach to collaboration	Guidance through clear structures
*“There’s still a lot of*,* like*,* I mean even in my company – they kind of don’t know the difference between a patient and a patient representative.” Code 29*,* Healthcare professional*	Uncertainty regarding the role of patient representatives	Expectations of the patient representative role
*“It’s about finding the right person*,* but it’s also quite simply about knowing where the boundaries are.” Code 16 Healthcare professional*	Role suitability as representative	Trust and communication
*“Patient representatives from loads of different organisations… on the premise*,* or like we’re going to share experiences…It never feels like this gets captured; it never feels like you get anything back*,* that you kind of*,* well. And then it feels a bit more like*,* this is kind of just ticking the box.” Code 19 Public authority representative*	Sharing experiences without the ability to influence	Towards shared understanding

### Research team and reflexivity

Reflexivity is considered a key component of rigour in qualitative research, emphasising the researchers’ active role in knowledge construction and the influence of their perspectives on the research process [38]. In this study, reflexivity was addressed through ongoing analytic discussions in which assumptions were critically examined and alternative interpretations explored.

The research team comprised researchers with diverse clinical and academic backgrounds, including a patient co-researcher, enabling complementary perspectives on the study. The team included a PhD candidate and registered nurse (KK), senior researchers with expertise in person-centred care, implementation, health innovation, and digitalisation (IB, AW, CW, ES), and a patient co-researcher (VPIN) with lived experience of the healthcare system. KK was involved in all phases, including design, data collection, analysis and manuscript preparation. The composition of the supervisory team (IB, AW, CW, ES) contributed expertise in person-centred care, patient involvement and healthcare development across clinical, academic and innovation contexts. VPIN contributed to data analysis and interpretation, drawing on lived experience to enhance the relevance of the findings. His academic background in gender studies and experience as a patient co-researcher ensured that the participant perspective remained visible throughout the analysis.

## Results

The analysis identified barriers and facilitators for PIE, which were grouped into the following themes: *Shifting Culture*,* Power*,* and Responsibility*; *Guidance through clear structures*; *Expectations of patient representative role*; *Trust and communication*; and *Towards shared understanding*. The ways in which these barriers and facilitators were understood and negotiated by different actors reflected underlying tensions related to differing (and sometimes conflicting) understandings of PIE and the factors that could influence its implementation.

### Shifting culture, power and responsibility

The analysis revealed a tension between viewing PIE as an important part of a broader societal shift in how involvement and engagement are understood and valued and the absence of consensus regarding who is responsible for advancing this shift. The development of PIE was seen as supported by political directives, as well as grassroots initiatives, with advocacy and lobbying identified as potential facilitators for advancing the agenda forward.

*…I’m thinking a little about those [patient] organisations working with policy*,* who want to be involved in changing society*,* er*,* or be involved and change Swedish health care and priorities and stuff like that…it’s a constant grind…and I think it’s that kind of influence that somewhere along the line puts pressure on decision-makers and others. (Code 20*,* industry representative)*

Despite this broader momentum, no clear consensus emerged about which key actors should take primary responsibility for moving the development forward, potentially constituting a barrier. Responsibility and power were frequently attributed to other key actors, with few perceiving their own group as primarily accountable. Different forms of power were mentioned in relation to PIE, such as economic power within the pharmaceutical/medical technology industry due to its financial resources.

*I feel the Med Tech companies have a greater…have a massive opportunity to get in touch. And I think a lot of it’s about the money…they’ve got the finances*,* so they have the time and that perspective anyway.” (Code 13, patient representative) *

Having direct access to patients, healthcare professionals were seen by other actors as holding significant power, for example, in relation to clinical trials. In contrast, patient representatives viewed researchers as influential due to their control over study design, their epistemic authority, and their command of scientific language. All key actors viewed the larger patient organisations as having a strong mandate and capacity with financial resources, established presence and societal goodwill.

*Patient organisations are a real driving force…with that and the other associations too*,* then you have quite a good reach*,* because it’s the big organisations that…that make a big noise. (Code 16*,* Healthcare professionals)*

Influence over PIE in research and development appeared to be structured around several distinct forms of power: economic power held by industry actors; access-based power associated with healthcare professionals; epistemic and scientific authority exercised by researchers; and organisational or representational power concentrated within large patient organisations.

### Guidance through clear structures

The participants expressed a clear need for shared guidelines and structures for PIE. At the same time, they recognised that actors operate across national, regional and global levels within diverse regulatory and institutional contexts. This creates a tension; while common frameworks are desired, the heterogeneity of actors and settings makes fully standardised guidelines difficult to implement. Participants also emphasised the importance of integrating PIE into everyday practice, pointing to a need for more formalised routines, clearer frameworks, and alignment with existing organisational processes. Such integration involved recognising the contextual and structural conditions that shape participation, including factors influencing patients’ ability to engage, such as time, resources, compensation, and digital accessibility. Taking these conditions into account may not only make involvement more feasible, but also more meaningful and equitable.

*… yes*,* it [PIE] really just needs to become something that is simply part of the process – that it shouldn’t be something exceptional*,* but rather the standard. And the path to get there is still quite long*,* even though we’ve come a long way already*… ( Code 29, Health Care professional)

Establishing supportive guidance, regulations and structures for collaboration were highlighted as an important means to advance the development of PIE practices and ensure a more equitable distribution of power and responsibility across key actors. To overcome this barrier, it was deemed necessary to develop a system or structure that facilitates interaction and contact, for example, through a digital platform. Representatives from the pharmaceutical/medical technology industry described having established systems, such as international patient databases, which enabled structured recruitment of patient representatives. However, unlike the other actors, industry’s scope as a global actor was seen as a barrier to collaboration with national or regional Swedish actors. Facilitating links between key actors could, for example, involve designating individuals responsible for identifying and engaging patient representatives, as well as fostering connections between patient representatives and other key actors.

A commonly identified barrier mentioned by several key actors was the lack of a shared and agreed-upon approach to PIE. Clear guidance defining the roles of involved actors and outlining how communication should take place between parties was described as a key facilitator for advancing PIE process. Such guidance was also seen as essential for clarifying the distribution of responsibilities and an important prerequisite for building trust and ensuring effective PIE. With more structured guidelines, patient representatives could become more equal and valuable partners in PIE processes. Patient representatives stated that it was confusing that the other actors each had their own frameworks and practices tailored to their specific institutional mandates and operational contexts. Collaboration between public authorities, patient representatives and the pharmaceutical/medical technology industry was described as particularly complex, shaped by diverging conditions and expectations. Patient representatives felt that they frequently had to adapt to conditions set by others and did not get access to collaboration unless they were invited by other actors. If invited, they did not always feel that they had any influence over how their contributions were acknowledged and utilised.

Unclear procedures for appointing patient representatives and a lack of transparency in nomination processes within patient organisations were also identified as barriers. The absence of shared criteria or agreed-upon guidelines for selection was described as contributing to uncertainty and inconsistency in representation.

*You have to meet and establish some criteria and guidelines and so on*,* and it’s a bit like what both need on the basis of what we have and think we have*,* etc. But there’s nothing like this currently as far as I know – currently we’re nominated by our respective associations (Code 10*,* patient representative)*

The findings indicate that PIE was marked by fragmented structures, unclear procedures and differing expectations across actor groups. Variations in institutional conditions, nomination practices and mechanisms for initiating collaboration shaped interaction in practice and contributed to unequal opportunities for engagement. Efforts to strengthen PIE were not understood solely as the introduction of standardised checklists or fixed routines, but rather as fostering a shared understanding among actors to guide how collaboration is carried out in practice.

### Expectations of the patient representative role

The findings revealed a tension concerning the expectations placed on patient representatives. On the one hand, they were expected to be well prepared, knowledgeable and capable of engaging in complex discussions alongside professionals. On the other hand, they were expected to retain their patient perspective without becoming shaped or influenced by other actors or by the context in which they were involved. This created a delicate balancing act, as increasing competence and familiarity with institutional processes risked positioning them as “too professional”– potentially distancing them from the very perspective they were invited to represent.

Participants also noted a shortage of available patient representatives, and that the same individuals were becoming highly visible across various forums to the extent that they were perceived as quasi-professionals. This raised questions about their ability to genuinely represent the broader patient population. Further, participants emphasised a need for new voices and fresh perspectives, arguing that broader inclusion is necessary. Some were also critical about “expert patients” who may not fully represent a diversity of patient experiences.

*Sometimes I*,* when I see all these debates*,* I do wonder…It’s the same people spinning around all the time. And sometimes I feel I’m turning into one of them too*,* and actually I usually point this out*,* especially in regard to regional patient representation – that it’s really important that we have a…That we change it because sometimes a fresh pair of eyes sees things. (Code 2*,* patient representative)*

Representativeness was a major issue, with all actors acknowledging that broad representation was important and should include what they understood to be marginalised groups. However, this created a tension with an idealised image of the “perfect” patient representative - an image articulated not only by patient representatives themselves but also by other key actors involved in PIE processes. Patient organisations were expected to provide representatives who were sufficiently trained while also able to navigate between their personal lived experiences and broader, collective insights related to a specific condition or diagnosis. They were expected to have formal training, familiarity with medical terminology, an understanding of processes of research and development, a broad general education and knowledge of the healthcare system, including relevant legislation and the national care guarantee.

*You kind of have to know a lot about health care in order to have an opinion. (Code 15*,* health care representative)*

A tension also emerged between the ambition to achieve broad representation and the high expectations placed on patient representatives. Insufficient attention was given to the individual circumstances and conditions shaping participation, including health limitations, inadequate accessibility and transport services, age-related factors, limited digital literacy and geographical or socio-economic disadvantages.

Another tension was that patient representatives were expected to be courageous and persistent in their advocacy, even in the face of rejection, while at the same time being constructive, realistic and solution-oriented. The ideal representative was expected to be both passionate, driven, attentive and empathetic while also acting with strategic awareness and purpose. Although lived experience was described as unquestionable and inherently valuable, it was considered important that the involvement of patient representatives extended beyond merely voicing complaints or recounting negative personal experiences. The role of the patient representative was framed as that of a *“carrier of voices*” for the wider patient community and as individuals from whom valuable input could be gathered and through whom feedback could be communicated back to the community.

*You have to be able to put yourself in someone else’s position*,* and be able to understand other people and clearly distance yourself from yourself*,* so to speak; and it’s not just you representing yourself and actually not yourself – it’s like the whole group. (Code 10*,* patient representative)*

The patient representatives felt they often needed to ‘prove themselves’ before being accepted as legitimate contributors, suggesting the existence of a threshold that needed to be crossed to gain recognition. Differing expectations, combined with practical constraints such as health-related limitations, accessibility issues, and unequal opportunities for participation contributed to a setting in which patient representatives were required to navigate complex and occasionally contradictory role requirements.

### Trust and communication

The findings revealed several interconnected tensions shaping trust and communication in PIE processes. A central issue raised by patient representatives was whether there was genuine interest in involving them, or whether involvement was primarily symbolic or tokenistic, and this was perceived as a barrier to meaningful engagement. Relationships between patient representatives and actors from the pharmaceutical and medical technology industry, healthcare and research were often described as characterised by mistrust, poor communication and ambiguity regarding roles and responsibilities.

*In the other regions we’ve had lots of invitations into contexts where we can participate…but if you are not [invited] and don’t know what you’re supposed to be doing – it becomes an obstacle. (Code 11*,* patient representative*)

Although intended to safeguard transparency and legitimacy, regulatory and ethical frameworks were in some cases perceived as constraining meaningful engagement.

*We want input on the data in clinical studies*,* say a phase three study*,* yes – but then we aren’t always permitted to communicate with patient representatives because the drug hasn’t yet been approved…and then it’s a catch 22. The patients want to participate*,* we want to hear what they think*,* if we’ve done something wrong*,* if there’s something we can do better…But that counts as impermissible marketing. (Code 23*,* industry representative)*

Patient representatives expressed a mistrust of the other actors in regard to whether they really found collaboration valuable or if they had hidden agendas that were not communicated. For example, funding agencies might demand PIE, which could make researchers feel forced to engage in it, or the pharmaceutical industry might use the involvement to promote a product. Public authorities faced a tension between the need to involve citizens for democratic reasons and the need to safeguard decision-making from undue influence. This was reflected in a reluctance to involve patients due to concerns about conflicts of interest, which in some cases risked making patient involvement superficial.

*Various authorities have been afraid of compromising impartiality and so on because they don’t want to listen to patients*,* because they want to keep an open – an open mind regarding the data and stuff like that. (Code 23*,* industry representative)*

The participants emphasised the importance of clarifying roles, expectations and conditions for participation, including issues related to compensation, something that can be understood in relation to the underlying tensions between expectations and practical conditions for involvement. A shared understanding of purpose, open communication, and clearly defined short- and long-term goals were seen as essential for building trust and preventing tokenistic involvement. Strengthening collaboration also required structure, communication skills, and long-term strategic efforts.

### Towards a shared understanding

The findings revealed a tension linked to differing understandings of what meaningful PIE involves and how involvement and engagement should be enacted. According to the pharmaceutical and medical technology industry and public authority representatives, collaboration was primarily seen as gathering input and perspectives or disseminating information to a patient group, thereby making the information more accessible to a broader patient population or the public. For health researchers, collaboration was most often related to engaging patients in clinical studies and ensuring their retention throughout the study period. Within the healthcare sector, collaboration was understood both as fostering relationships at the individual level and as collecting feedback on patients’ journeys through the healthcare system. Patient representatives often expressed goals for collaboration that differed from those of other key actors, their aim being not only to improve their own situation but to advocate for change that would benefit their entire patient group. These differing understandings highlight a fundamental tension in the purpose and scope of PIE, which may lead to misaligned expectations, challenges in collaboration, and a risk of involvement being perceived as limited or tokenistic.

*Being a civil society organisation representing members or patients is something different. It has its own logic and its own integrity. I think that we*,* as patient representatives and civil society organisations*,* could become better at safeguarding that integrity. But I also think that the professions and academia could become better at understanding our logic. (Code 7*,* patient representative)*

Patient representatives often characterised their role as primarily consultative and questioned whether their contributions translated into actual influence over decisions. This appeared to be partly related to a lack of feedback regarding how their input had been used and partly to what it had led to, which further reinforced perceptions of limited influence. Moreover, several key actors highlighted the risk of collaboration becoming tokenistic or reduced to a box-ticking exercise as a barrier and contrasted this with the notion of meaningful involvement.

*Patient representatives from loads of different organisations…are invited in on the premise*,* or like we’re going to share experiences…It never feels like this gets captured; it never feels like you get anything back*,* that you kind of*,* well. And then it feels a bit more like*,* this is kind of just ticking the box. (Code 19*,* Public Authority representative)*

These findings highlighted that strengthening PIE requires a shared understanding of collaboration amongst all actors, including patient representatives, healthcare professionals, health researchers, the pharmaceutical and medical industry, and public authorities. Developing approaches that clearly communicate the nature of collaboration, its expectations, and the roles of patients was viewed as key to strengthening PIE.

## Discussion

This study aimed to examine how barriers and facilitators to PIE in health research and development are understood and negotiated by different key actors. While the study identified barriers and facilitators similar to those reported in previous research, it also highlighted tensions between participants’ different understandings of barriers and facilitators, and how these were interpreted and negotiated across different contexts.

One of these tensions concerned how the role of the patient representative was understood differently by different actors. Although everyone had high expectations of patient representatives, some of these expectations were almost contradictory, making the role difficult to navigate. Patient representatives were expected to share their personal lived experience while simultaneously being highly knowledgeable about research and organisational processes and representing broader patient groups. Previous research shows that patient representatives are often expected to move beyond personal experiences representing collective perspectives [39, 40]. Ethical analyses have also questioned the extent to which patients are expected to advocate within systems where decision-making power largely remains with professionals [41]. Another tension was related to the discrepancy between the high value ascribed to PIE and patients’ lived experiences, and the limited financial and structural support provided to patient representatives. Although the value of PIE was widely acknowledged, patient representatives described challenges previously reported in the literature, including high expectations combined with limited or no financial compensation. This places additional burdens on individuals already managing limitations related to their illness and life circumstances and continues to shape how PIE activities are implemented in practice [2, 42, 43]. Such role ambiguity may shift responsibility onto patient representatives while the conditions for PIE remain controlled by other actors. Our findings reflected previously described power asymmetries between patient representatives and other actors, including differences in influence and access to the decision-making arena, as also highlighted in earlier research [2, 44]. What this study adds is that no single actor appeared to acknowledge their own power. Instead, power was often attributed to others, resulting in a diffusion of responsibility that complicated efforts to advance PIE. Such diffusion of responsibility was also reflected at a structural level. Despite a growing commitment to PIE and increasing recognition of the value of patient perspectives, participants described considerable ambiguity regarding who held responsibility for advancing involvement. Responsibilities frequently shifted between actors, with organisations developing their own structures to manage PIE within their specific contexts. Consequently, a tension was felt between collective endorsement and fragmented ownership, whereby support for PIE is widely expressed but governance remains dispersed. Such fragmentation may limit the potential for PIE to become systematically embedded in health research governance and risks leaving involvement dependent on individual initiatives rather than coordinated structures. Co-design approaches may offer a way to address this diffusion of responsibility by fostering shared ownership and more explicit negotiation of roles and influence. Co-design approaches emphasise the importance of sharing power between researchers and patients and recognising experiential knowledge in the development of healthcare interventions [45]. Thus, in view of the above combined with the findings of our study, strengthening PIE would appear to require not only including patient representatives but also actively addressing how power is distributed in involvement processes. This may involve creating structures that support more equal participation, clarifying roles and expectations, and ensuring that patient perspectives can meaningfully influence decisions. Such structures may involve allocating time and resources and clarifying roles and expectations that foster a more collaborative process [46].

Previous research has highlighted governance challenges, emphasising that meaningful PIE requires not only structural adjustments but also broader cultural change across organisations, including shifts in organisational processes and mindsets [47–49]. Studies have also pointed to the lack of clear conceptual frameworks and coordinated structures for PIE at meso- and macro-levels of health systems [2, 23]. In addition, patient advocacy, political directives, and grassroots initiatives have been identified as important drivers in shaping research agendas and advancing involvement [49]. Although participants in this study expressed a need for more shared structures to support PIE, this also revealed an inherent tension, as establishing uniform approaches across actors may be challenging given the diversity of organisational contexts, logics, responsibilities, and institutional conditions in which they operate. Meaningful collaboration therefore depends on recognising and negotiating these differences. Instead of imposing standardised models, it is important to develop and co-create approaches to PIE that are grounded in shared principles and expectations, while remaining sensitive to contextual variation in practice. In this sense, advancing PIE is not only a matter of developing structures, but also of aligning perspectives and conditions for collaboration through co-creation in ways that are mutually understood and collectively sustained [50, 51].

Another tension concerned how PIE was understood and negotiated across different actors. Although PIE was described as a shared concept, its meaning varied depending on context. For example, representatives from the pharmaceutical and medical technology industry and public authorities mainly described PIE as a way to gather patient input or disseminate information, whereas health researchers associated it with engaging and retaining patients in clinical studies. Within healthcare, PIE was understood both as building relationships at an individual level and as collecting feedback on patient experiences, while patient representatives emphasised broader aims of improving conditions for their patient group and advocating for better healthcare. Consequently, there is a tension between a shared concept and differing interpretations in practice, which may create uncertainty about the aims of involvement and contribute to mistrust and concerns about hidden agendas. Previous research has highlighted similar barriers, emphasising that PIE is often interpreted and implemented differently across organisational and institutional contexts [2, 5, 23]. These studies suggest that ambiguity around definitions and purposes can create barriers to collaboration and shared expectations in involvement processes. However, the participants in this study also highlighted that achieving complete consensus on definitions may be less important than ensuring open communication and transparency between actors. By clearly articulating the purpose, roles, and expectations related to PIE, actors may develop a shared understanding even when their interpretations and sometimes goals differ, thereby reducing ambiguity and supporting more meaningful collaboration. This is also highlighted in previous research, which shows that collaborative practices in health services research often involve actors with differing perspectives, institutional logics, and priorities [52, 53]. Despite these differences, shared concepts and practices may enable coordination between actors while allowing for multiple interpretations of their meaning and purpose. Thus, PIE can be understood as a boundary object, referring to a concept or practice that provides a shared point of reference across different contexts while remaining open to diverse interpretations [54, 55]. Effectiveness of boundary objects depends on how they are interpreted, adapted, and enacted in specific contexts. This involves processes of co-production and ongoing adaptation [56], as well as active facilitation to support translation, relationship-building, and alignment across actors [55, 57].

In the present study, the Swedish healthcare system formed the contextual point of departure, which may differ from systems in countries where PIE has been more extensively developed. Patient organisations may vary in how they are formed, funded, and organised in Sweden compared to other countries, which may influence how PIE activities are implemented. In addition, Sweden’s 21 self-governing regions play a key role at the meso level of the healthcare system, resulting in variations in how patient involvement is organised and implemented across regions, while also creating opportunities for local innovation [27, 58]. Previous research suggests that patient involvement in Sweden has often been limited in scope and impact, and that citizen dialogues frequently produce unclear outcomes [27, 59]. At the same time, experiences from co-producing clinical pathways across all 21 regions demonstrate opportunities for more systematic forms of engagement [60].

An important question arising from these findings is how to address the tensions identified in this study to support the further development of PIE. In this regard, the principles of a person-centred approach may offer a useful framework for addressing several of the challenges highlighted. For example, while there is a clear call for guidance through structures, standardised approaches to PIE may be difficult to apply given the diversity of actors, contexts, and perspectives involved. Rather than seeing PIE through the lens of a predefined model, it can be understood as a relational practice in which actors negotiate common ground despite divergent perspectives. A person-centred approach thus offers a relevant framework, as its emphasis is on narrative, partnership, and shared responsibility trough documentation. It may lay a foundation for more collaborative relationships and more coherent involvement practices [61, 62].

Implementing PIE through a person-centred approach can be advanced in three interconnected steps: *establishing the partnership through a shared narrative*, *enacting the partnership through explicit negotiation of roles and decision-making responsibilities*, and *sustaining the partnership through continuous documentation and follow-up*. Collectively, these steps provide a structured and adaptable framework for embedding person-centred principles in PIE (and PPI) while fostering trust over time (J. Bergholtz et al., unpublished commentary, 2026). Our analysis indicates that these steps would represent a flexible framework that may be transferable across contexts, forms of involvement, the actors involved, and different levels of healthcare systems. To support such approaches, organisations need to support long-term collaboration between actors. This may include setting aside time and resources for partnership-based collaboration, clarifying roles and responsibilities, and creating routines for ongoing dialogue and follow-up. At the policy level, PIE needs to be integrated into organisational strategies and governance rather than being carried out as separate or temporary activities. A person-centred approach could thus be fostered by supporting more collaborative and meaningful patient involvement in both health research and service development. To facilitate this at the organisational level, certain fundamental conditions are required, described by Bergholtz et al. [63] as *pre-conditions for* person centred*-PPI*. These include *ethical-capability readiness*, *structural-material readiness*, and *governance-accountability readiness*. Ethical capability refers to reciprocal capacity-building, whereby both professionals and PIE contributors develop the relational, communicative, and ethical competencies needed for genuine and sustainable collaboration. Structural-material readiness concerns whether organisations provide the resources, accessibility, and administrative support necessary to enable equitable and sustainable PIE. Governance-accountability readiness refers to organisational arrangements that make the influence of PIE visible, accountable, and open to evaluation over time.

Framing PIE through a person-centred approach may support a shift from procedural involvement towards a more transformative practice that shapes research priorities, organisational processes, and policy decisions in line with patient and public values.

### Strengths and limitations

A key strength of this study was the involvement of a patient co-researcher throughout the research process, particularly during the analysis and manuscript development, which strengthened the visibility of the patient perspective. The inclusion of participants from several key actor groups involved in patient and public involvement and engagement (PIE) also enabled exploration of diverse perspectives on barriers and facilitators across organisational contexts.

However, some limitations should be considered. Participants were recruited through EUPATI, ensuring familiarity with PPI/PIE and enabling in-depth discussions, but this may also have introduced selection bias. In addition, the distribution across actor groups was uneven, with patient representatives more strongly represented than other actors, such as health researchers, healthcare professionals, public authorities and the pharmaceutical/medical technology industry. Data collection through semi-structured Zoom interviews allowed for geographically broad participation, although the format limited the observation of non-verbal communication.

Trustworthiness was ensured in accordance with Graneheim and Lundman’s criteria of credibility, dependability, and transferability [29, 30]. Credibility was strengthened through the use of semi-structured interviews, allowing participants to elaborate on their experiences. The analysis involved iterative discussions within the research team to reach consensus on codes and categories grounded in the data. Dependability was supported by a transparent and systematic analytical process, in which the steps were documented and discussed within the team. Transferability was facilitated by providing detailed descriptions of the study context, participants, and analysis.

## Conclusion

The findings demonstrate that barriers and facilitators to PIE are not fixed but are continuously negotiated between actors within specific organisational and institutional contexts. These negotiations are shaped by power relations, role expectations, and access to decision-making, often placing disproportionate responsibility on patient representatives while their influence remains constrained. At the same time, a tension emerges between the demand for clearer structures and guidance and the practical challenges of standardising PIE across heterogeneous contexts.

Addressing this requires the development of shared yet flexible frameworks that clarify expectations while remaining sensitive to contextual conditions. Integrating person-centred principles into PIE may offer a way forward and help address these tensions. Future research should further examine how such negotiated practices can support more equitable and impactful forms of involvement, as well as how person-centred approaches may contribute to addressing these challenges. 

## Supplementary Information

Below is the link to the electronic supplementary material.

Additional file 1: COREQ checklist. Description: This file provides the completed COREQ (Consolidated Criteria for Reporting Qualitative Research) checklist used to ensure comprehensive and transparent reporting of the qualitative interview study

Additional file 2: GRIPP2-SF reporting checklist. Description: This file provides the completed GRIPP2-Short Form (Guidance for Reporting Involvement of Patients and Public) checklist, documenting how patient and public involvement was integrated into the design, conduct, and reporting of the study. The checklist ensures structured, transparent, and comprehensive reporting of all PPI activities

Additional file 3: Interview guide. Description: This file contains the full semi-structured interview guide used during data collection, including all main questions and prompts provided to participants

Additional file 4: Overview of codes and themes. Description: This file provides an overview of the codes generated during the analytic process and how these were grouped into themes. The table presents the final set of themes identified in the analysis together with the associated codes that contributed to their development. The coding process was iterative, and the codes were continuously discussed and refined within the research team before being organised into the themes presented in the Results section

## Data Availability

The datasets generated and analysed during the current study are not publicly available due to ethical and legal restrictions under Swedish data protection regulations but are available from the corresponding author on reasonable request and with appropriate ethical approvals.
