# Supplementary material for: Tensions in patient involvement and engagement in health research and development: a qualitative interview study with key actors
Source: Res Involv Engagem. 2026 Jun 11;12:87. doi: 10.1186/s40900-026-00915-3 (PMC13255309; doi:10.1186/s40900-026-00915-3)
Supplement: Supplementary file 2 — Additional file 2: GRIPP2-SF reporting checklist. Description: This file provides the completed GRIPP2-Short Form (Guidance for Reporting Involvement of Patients and Public) checklist, documenting how patient and public involvement was integrated into the design, conduct, and reporting of the study. The checklist ensures structured, transparent, and comprehensive reporting of all PPI activities [file 40900_2026_915_MOESM2_ESM.pdf]

Appendix A. GRIPP2 Reporting Checklist – Short Form (Staniszewska et al., 2017)

Section and topic	Item	Reported on page
1: Aim<br>Report the aim of PPI (patient and public involvement) in the study	The aim of patient and public involvement (PPI) in this study was to ensure that the patient perspective was integrated throughout the research process, from the analysis of data to the final manuscript. PPI served as a bridge between the experiential knowledge of patients and the academic analysis provided by researchers. Details of this involvement are presented under *Methods* and *Strengths and Limitations*.	Page 7
2: Method<br>Provide a clear description of the methods used for PPI in the study	A patient co-researcher (VPIN) was actively involved in all phases of data analysis. He participated in interpretative discussions, contributed to theme refinement, and reviewed the final manuscript draft to ensure clarity and relevance from a patient perspective.	Page 7
3: Study result<br>Outcomes: report the results of PPI in the study, including both positive and negative outcomes	Drawing on lived experience, the patient co -researcher helped contextualise findings and bridge perspectives between researchers and patients. Their input led to revisions in the way results were framed and presented.	Page 7
4: Discussion and conclusions<br>Outcomes: comment on the extent to which PPI influenced the study overall. Describe positive and negative effects	The patient co -researcher contributed substantively to the discussion and conclusion sections of the manuscript, ensuring that the implications of the findings were grounded in the patient experience. This collaboration contributed to more relevant interpretations and enhanced the study's trustworthiness and applicability. No major negative effects were identified, although the process highlighted the need for clearer role definitions early in the project.	Pag 28
5: Reflection/Critical perspective<br>Comment critically on the study, reflecting on the things that went well and those that did not, so others can learn from this experience	Patient co-researcher was mainly involved in the analysis and manuscript writing. His input enriched the interpretation of the findings and improved the clarity and relevance of the results. Clear communication and defined roles supported effective collaboration, and future projects could benefit from involving a patient co-researcher earlier in the research process.	Page 28

PPI= Patient and Public Involvement
