# Supplementary material for: Tensions in patient involvement and engagement in health research and development: a qualitative interview study with key actors
Source: Res Involv Engagem. 2026 Jun 11;12:87. doi: 10.1186/s40900-026-00915-3 (PMC13255309; doi:10.1186/s40900-026-00915-3)
Supplement: Supplementary file 3 — Additional file 3: Interview guide. Description: This file contains the full semi-structured interview guide used during data collection, including all main questions and prompts provided to participants [file 40900_2026_915_MOESM3_ESM.pdf]

Introductory Questions

- Can you tell me a bit about yourself and your role today?
  What do you work with and what do you do in that role?

Patient Involvement and Engagement PIE (general)

- How would you describe PIE to someone who doesn't know what it is?
  What does PIE mean to you?
  What risks do you see when it comes to PIE?
  What opportunities do you see with PIE?
- Can you give examples of what PIE is in practice based on your experience?
  Can you give examples of what PIE is in a situation in collaboration with other key actors?
  How is PIE relevant in your work? Can you give examples of specific situations?
  Which key actor do you collaborate with the most?
  Do you collaborate with patients or patient associations?

Patient Involvement and Engagement (Specific Situation)

- How do you perceive the treatment of patient/relative representatives?
  What do the terms patient representative/relative representative mean to you?
  What would you say is good treatment of a patient representative?
  Can you describe a situation where a representative received good treatment?
  Can you describe a situation where a representative received poor treatment?
  What do you consider important in the meeting with the patient/relative representative?
- Can you give examples of what hinders PIE?
- Can you give examples of what enables PIE?

Influence/Impact on Patient Involvement and engagement

- How do you perceive the influence of patient/relative representatives?
- Which key actors do you think have the greatest ability to influence or determine the degree of PIE?
  Can you describe a situation where a patient representative had a lot of influence?
  Can you describe a situation where a patient representative had little influence?
  Can you, in your role, influence the impact of patient and relative representatives?
  Have you been in a situation where a representative had too much/little influence?
- Which actor do you think has the greatest ability to influence how PIE happens in practice?
  A lot or a little, what is enough?
