# Supplementary material for: Tensions in patient involvement and engagement in health research and development: a qualitative interview study with key actors
Source: Res Involv Engagem. 2026 Jun 11;12:87. doi: 10.1186/s40900-026-00915-3 (PMC13255309; doi:10.1186/s40900-026-00915-3)
Supplement: Supplementary file 4 — Additional file 4: Overview of codes and themes. Description: This file provides an overview of the codes generated during the analytic process and how these were grouped into themes. The table presents the final set of themes identified in the analysis together with the associated codes that contributed to their development. The coding process was iterative, and the codes were continuously discussed and refined within the research team before being organised into the themes presented in the Results section [file 40900_2026_915_MOESM4_ESM.pdf]

Theme	Shifting Culture, Power and Responsibility	Guidance through clear structures	Expectations of the patient representative role	Trust and communication	Towards shared understanding
Code	1.Ambiguity in power relations in the context of collaboration<br><br>2.Responsibility placed on someone else<br><br>3. Epistemic authority<br><br>4. Link between different actors<br><br>5. Self-awareness of one's own power<br><br>6. Support from the organization<br><br>7. Size of the organization<br><br>8. Shift in power perceived<br><br>9. Everyone holds power<br><br>10. Language as power	1.The importance of a systematic approach to collaboration<br><br>2.Hierarchies in systems and relationships<br><br>3.Identifying key actors<br><br>4.Democratic governance<br><br>5.Lack of transparency<br><br>6.Participation at micro, meso and macro level<br><br>7.Knowledge transfer, if it occurs<br><br>8.National guidelines<br><br>9.International perspective<br><br>10.Models for PPI to follow	1.Uncertainty regarding the role of patient representatives<br><br>2.The same individuals circulating and representing<br><br>3.Too knowledgeable to be considered legitimate<br><br>4. Representing oneself or a group<br><br>5.Expectations from others<br><br>6.Must have courage as a patient representative<br><br>7. Solution-oriented<br><br>8. Seeking knowledge<br><br>9. Being unsuitable as a patient representative<br><br>10. Security through experience<br><br>11. Expectations of formal training and system knowledge	1. Role suitability as representative<br><br>2.Different purposes of PIE<br><br>3.Different definitions<br><br>4. Shared goals<br><br>5. Participation through dialog<br><br>6. checkbox approach<br><br>7. Outcomes of participation – receiving feedback on involvement<br><br>8. Communication<br><br>9. The patient perspective<br><br>10.Knowledge in healthcare	1.Sharing experiences without the ability to influence<br><br>2. Perceived responsiveness to patient input<br><br>3. degree of participation<br><br>4.Early involvement<br><br>5. Clarity and transparency in collaboration<br><br>6. Mutual knowledge exchange through dialogue<br><br>7. Building relationships between different actors<br><br>8. Respect for different institutional logics<br><br>9. Treatment based on the current role
